# Image-Based vs. Parametric Modelling of Concrete Meso-Structures

**DOI:** 10.3390/ma15030704

**Published:** 2022-01-18

**Authors:** Jiaming Wang, Andrey P. Jivkov, Dirk L. Engelberg, Qingming Li

**Affiliations:** 1 Department of Mechanical, Aerospace and Civil Engineering, University of Manchester, Manchester M13 9PL, UK; Qingming.Li@manchester.ac.uk; 2 Materials Performance Centre, Department of Materials, University of Manchester, Manchester M13 9PL, UK; D.Engelberg@manchester.ac.uk

**Keywords:** meso-scale, concrete damage plasticity model, cohesive zone model, zero-thickness ITZ, X-ray computed tomography, quasi-static loadings, energy dissipation

## Abstract

Damage initiation and crack propagation in concrete are associated with localisation of energy dissipation by the concrete meso-structure. Meso-scale models are, therefore, required for realistic analysis of concrete non-linear behaviour. Such models are constructed either from X-ray Computed Tomography images (image-based modelling) or by in silico meso-structure generation (parametric modelling), while both approaches are widely used and their advantages and disadvantages are recognised, little work is done on comparing their performance in predicting measured macroscopic behaviour with equivalent constitutive relations for meso-structural features. This work uses microstructure characterisation and mechanical behaviour data to construct, validate and compare the two modelling approaches. The macroscopic behaviour obtained with both meso-structural models is found to be in good agreement with experimental data. Differences are observed only between the predicted distributions of damage within specimens. These outcomes suggest that the computationally simpler parametric meso-structures are sufficient to derive stress–strain behaviour for engineering-scale models in the absence of other environmental factors. The observed differences in damage distribution could be important for analysis of coupled behaviour, e.g., mass transport and chemical reactions affecting local mechanical properties and being affected by local damage. Establishing the importance of damage distribution is such cases requires further research.

## 1. Introduction

The macro-cracks of concrete are developed through micro-crack initiation, propagation and coalescence. The reliable prediction of concrete component failure requires in-depth understanding of the localized crack evolution of concrete heterogeneous composition. Compared with the homogeneity at macro-scale, meso-scale concrete consists of heterogeneous phases, including coarse aggregates, mortar (cement paste with sand and fine aggregates embedded) as matrix, and entrapped air voids. Interfacial transition zone (ITZ) is not observable at meso-scale, but provides both preferable locations for crack initiation and easier pathways in the damage evolution. ITZ has lower stiffness and strength compared with mortar, because it is a thin layer of higher-porosity mortar coating around aggregates with thickness between 10 and 100 μm [1,2].

The aggregate distribution of meso-scale concrete can be obtained by digital image acquisition of realistic size and location of aggregates or by random spatial distribution of aggregates of given shapes with prescribed size distribution. As a non-destructive imaging method, X-ray computed tomography (XCT), has been widely used for acquisition of concrete meso-structures [3,4,5]. Different phases can be identified by threshold of grey value pixels. The 2D images obtained from XCT can be processed and reconstructed into a 3D model, which can be further meshed in the commercial software Simpleware [6] or self-developed algorithm [5,7]. The latter approach is to generate coarse aggregates synthetically based on given size distribution. This can be achieved by take-and-place method [8,9,10,11,12] and Voronoi tessellation method [13,14,15,16,17]. In the take-and-place method, aggregate particles are ’taken’ from a source following a certain size gradation and ’placed’ one by one into concrete model without overlapping with existing particles [10,11,12]. In the process of placing aggregates, they can be translated and rotated randomly in order to achieve a high aggregate volume fraction (e.g., >50%) [18,19].

The advantage of image-based models is that accurate geometry of aggregates and voids can be obtained. However, the image processing is time-consuming and usage of XCT equipment is expensive. Moreover, the imaged regions may not be statistically representative in terms of size and spatial distribution of aggregates. On the other hand, parametric models have the capacity to cover larger engineering component volumes statistically, where aggregates with presribed size distribution and volume density can be included. The limitation is that the randomly generated aggregates differ from the natural ones of the tested specimen in terms of both position and shape. One approach for using the XCT images in constructing synthetic models is to replace natural aggregates observed by XCT with circular ones of equivalent volumes at the same locations [20,21]. Such a process may lead to overlapping circular aggregates, which requires further adjustments. Nevertheless, it has been shown that there is no significant difference in the predicted stress–strain behaviour by the XCT-informed synthetic models and image-based models. However, the so created synthetic models inherit the size limitation of the image-based models. It is of clear practical interest to establish whether a fully parametric model, i.e., with random spatial distribution of aggregates with prescribed volume fraction and size distribution, can predict reliably the macroscopic behaviour.

In most of previous models, as well as in the present work, aggregates have been assigned with elastic behaviour, because their strength is higher than concrete failure stresses. Micro-cracks initiate in ITZ and propagate through mortar, which form macro-cracks in concrete structure. Mortar has been considered as inelastic homogeneous continuum with elastic-damage [5,12,13,22,23,24], elastic-plastic [24,25,26,27] or elastic-plastic-damage [7,24,28,29,30] behaviour.

The appropriate representation of ITZ is mostly debated. The first option is to neglect ITZ and attribute inelastic behaviour of concrete to mortar via elastic-plastic [25,26,27] or elastic-plastic-damage [28] constitutive behaviour. However, it is found to over-estimate concrete compressive strength [31]. Some researchers have generated a layer of finite thickness solid/continuum elements surrounding aggregates to represent ITZ, where the elastic-plastic-damage behaviour of mortar exhibit higher strength than that of ITZ. However, the layers used in this approach have significantly larger thickness than the physical ITZ thickness due to the mesh complexity and computational cost of using realistic thicknesses [7,28,29,30]. The third option is to generate zero-thickness cohesive elements (CE) between mortar and aggregates as ITZ. In some cases, zero-thickness CE have been applied between mortar elements. In such case, these elements are assumed to be elastic and inelastic behaviour of concrete depends on damage of CE at ITZ and within mortar [5,12,13,22,23,32]. If zero-thickness CE are only adopted at ITZ, mortar elements are assigned with plastic-damageable behaviour and ITZ CE are damageable [24,31,33,34,35]. Wang et al. [33] have extended the plastic-damageable mortar behaviour proposed by Unger et al. [24], considering both tensile and compressive hardening variables, which can be calibrated by tensile and compressive tests with mortar specimens. Furthermore, Wang et al. [31] have compared different ITZ representations (i.e., without ITZ, with finite thickness ITZ and with zero-thickness CE for ITZ). It has been found that the last representation can balance the physical realism of the concrete meso-structure model with computational efficiency, while providing predictions for stress–strain behaviour and damage evolution in agreement with experimental data. Therefore, in current study, meso-scale concrete is modelled with experimentally calibrated plastic-damageable mortar and zero-thickness cohesive elements as ITZ.

Parametrically generated meso-structures are appropriate for covering regions of large-scale engineering structures where damage/fracture is expected to initiate due to stress concentrators. This cannot be accomplished with image-based meso-structures due to the limited sizes of the CT scanned specimens. It is therefore necessary to understand how reliable the parametric meso-structures are in predicting the macroscopic response of concrete so that they can be used in analysis of large-scale engineering structures. The aim of this work is to established the areas of applicability of the two approaches for meso-structure construction, image-based and parametric/synthetic, by comparing their predictions for deformation behaviour and damage, supported by experimental data. One novel element of the work is the efficient segmentation of concrete constituents using artificial intelligent based segmentation tool—a trainable Weka segmentation plugin of ImageJ. Comparisons with own experimental data show that the synthetic meso-structures are sufficient for predicting the macroscopic stress–strain behaviour. However, damage is shown to develop differently in different meso-structures, suggesting that non-mechanical processes that depend on damage, such as diffusion and chemical reaction, require further investigation to demonstrate reliability of parametric models.

## 2. Experimental Setup

### 2.1. Materials

According to British Standard [36], the C30 grade concrete specimens were designed with CEM I 42.5 Ordinary Portland Cement (OPC) and coarse limestone (diameter between 6.3 and 10 mm), as shown in Table 1. Concrete specimens with three aggregate volume fractions, i.e., 20%, 30% and 40%, were prepared. Both concrete and mortar specimens have a fixed w/c ratio of 0.49. It should be noted that the sand content of mortar is the same with that of concrete for each aggregate volume fraction, but the three types of mortar has different compositions because of the constant designed concrete strength. Uniaxial compression experiments were carried out on cylindrical specimens of diameter 100 mm and height 200 mm, and on cubical specimens of dimension 50 mm specimens, while uniaxial tension experiments were carried out on dogbone specimens of length 90 mm and thickness 25 mm. All specimens were cast in moulds, removed from moulds after 24 h, and cured in a water tank for 28 days before testing.

**Figure 1:**
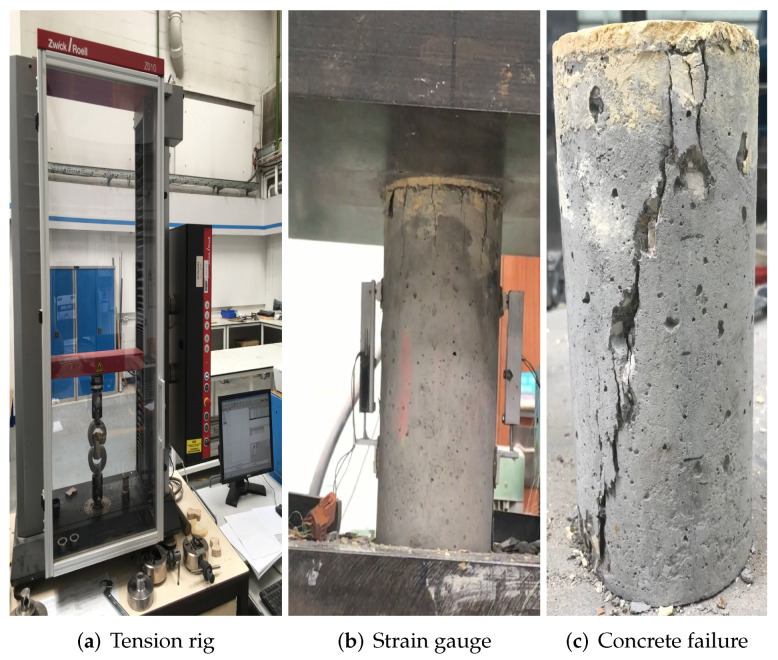
Experimental set up [31,34].

The test rig and different specimen types used are shown in Figure 1. Three specimens of each geometry and aggregate volume fraction were tested. Uniaxial compression tests were performed on Amsler compression machine with a loading rate of 0.2 MPa/s, where strain gauges with precision of ±0.1% were mounted on the surface of the specimens. Uniaxial tension tests were performed on the universal testing machine with a loading rate of 0.2 mm per minute, where linear variable differential transformers were used to record the displacements. Only one or two data points were useable for the post-peak behaviour in compression, due to the lack of displacement control. No tensile post-peak data was recorded, because the dogbone specimens failed instantly. Cylindrical and dogbone specimens of mortar were used to derive the parameters of mortar damage-plasticity constitutive law. These parameters are given later in Table 2. Experiments with concrete specimens were used for models validation; values are reported later in Table 3. It should be noted that the simulations under tensile load described later in the work use cylindrical volumes, which is different from the experimental setup with dogbones. This was done to reduce computational cost.

### 2.2. X-ray Computed Tomography Technique

**Figure 2:**
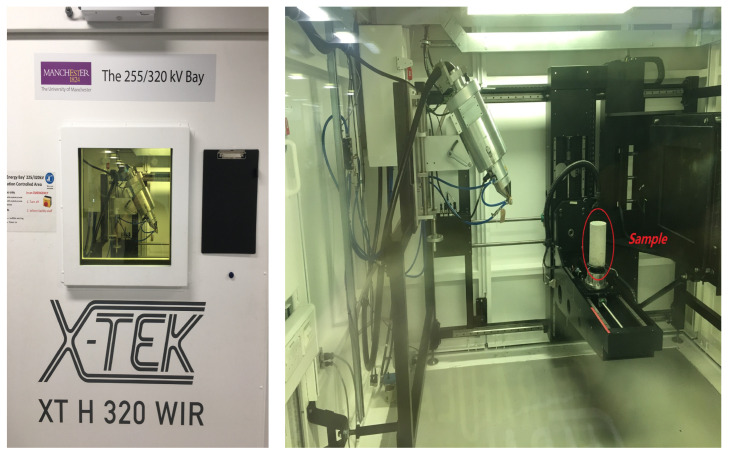
Nikon XTH 225 custom bay in Henry Moseley X-ray Imaging Facility.

The X-ray Computed Tomography (XCT) scanning was carried out prior to mechanical testing on one cylinder of each aggregate content at the Henry Moseley X-ray Imaging Facility, the University of Manchester with the Nikon XTH 225 (Nikon, Tokyo, Japan) custom bay (see Figure 2). The XCT scanner was set up with an exposure time of 1 s at an accelerating voltage of 220 kV and 111 μm beam current. A 0.004 mm copper filter was also used. The stage rotated 360 degrees during each scan and 2000 projections with voxel size of 0.098 mm were collected. Software CT Pro (version 2.0) was used to process 2D images and reduce beam hardening. These images were further reconstructed into 3D models in AVISO software (version 2019) package for visualisation and phase segmentation.

**Figure 3:**
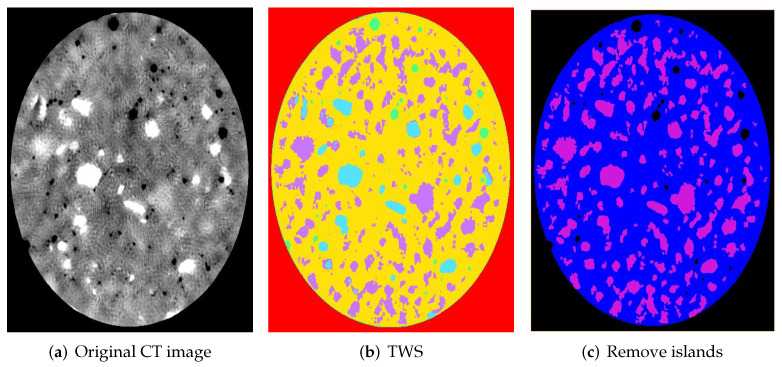
XCT image after processing and segmentation. Limestone area: (**a**) grey, (**b**) purple and (**c**) purple; Sand (or small stone) area: (**a**) white, (**b**) blue and (**c**) purple.

**Figure 4:**
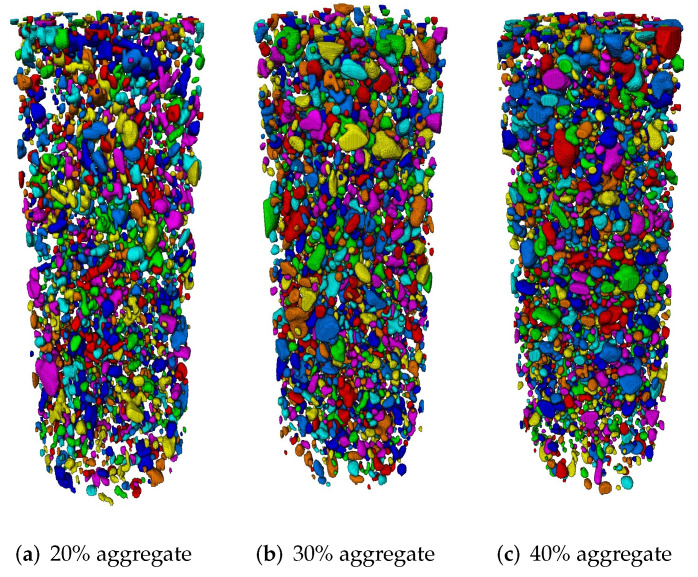
Segmented aggregates of models from XCT images with (**a**) 20%, (**b**) 30% and (**c**) 40% aggregate content.

The core (D50*100 mm) was cropped from the whole cylinder (D100*200 mm) to reduce data processing time and edge effect. Non-local means filter was applied to remove noise and unify grey value in different phases. Due to the enormous amount of aggregates in 2000 slice images, the trainable Weka segmentation (TWS) plugin was adopted in software Fiji (ImageJ, version 1.52), which can segment selected image features based on machine learning algorithms. The TWS plugin can automatically identify the regions of interest when the users define different phases and mark the identical features in each phase. After several trainings, TWS plugin can identify almost all of the regions of interest, as shown in Figure 3a,b. The sand (or small stone) phase of Figure 3b is coloured in blue and the limestone is coloured in purple. Note that the cross-section area may not reflect the actual size of aggregates. Both sand and limestone were considered as coarse aggregates during segmentation, but those smaller than 5 mm were removed and included in the mortar at final step of segmentation. After removing islands of certain voxel size, noise on segmented images was reduced (see Figure 3c). Furthermore, the connection of aggregates was checked manually compared with the original CT image. Figure 4 shows the segmented aggregate particles of concrete models with three aggregate contents. Each separate aggregate was labelled with a different colour.

Statistical analysis of each phase was carried out. The aggregate volume fractions of image-based concrete were found to be 19%, 33% and 39% respectively, in agreement with the concrete mix designs of 20%, 30% and 40% aggregate content. The porosities of the three mixtures were found to be 1.4%, 1.3% and 0.9%, respectively. Most of the voids were between 2 and 4 mm in diameter. Notably, the voids observed on the outer surface of the lab cast concrete cylinders, were not replicated in the parametric concrete meso-structures. Therefore, the porosity of parametric models was set to be 1% for all three aggregate contents.

## 3. Meso-Structure Generation and Meshing

The image-based meso-scale concrete model was obtained after XCT scanning, 3D reconstruction and phase segmentation. The other approach to generate concrete meso-structure is via synthetic parametrization. Aggregate particles are randomly distributed in the concrete volume without overlapping each other, but follow the designed aggregate size distribution. Although the shape and location of synthetic aggregates do not correspond to a particular real meso-structure, the potentially different macroscopic behaviours can be assessed by Monte Carlo simulations [37]. Coarse aggregate and air voids are generated with spherical shapes, because their shape effect on concrete mechanical behaviour is insignificant in the pre-peak regime [38]. Meso-scale concrete cylinders with 50 mm diameter and 100 mm height are generated for tension and compression simulations.

The take-and-place method to generate the randomly distributed aggregate and pore particles is similar to Wang et al. [12], where a detailed algorithm of “input”, “taking” and “placing” steps can be found. First, particle parameters in terms of the size and shape distribution were recorded in the “input” step. Next, individual particles with random size within the prescribed distribution are generated in the “taking” step, and the aggregate particles are generated before air voids. In the “placing” step, the generated particle is located in the random position of the domain volume if its overlapping and intersecting conditions with existing particles and volume boundaries are satisfied. Finally, the volume fraction of generated particles is calculated. If prescribed value is satisfied, the generation procedure terminates.

The meshing of concrete meso-structures obtained from XCT scan and generated by the take-and-place method was performed with common approach, by tessellating the domain into voxels and generating finite element mesh in the commercial code ScanIP (Simpleware Ltd., London, UK) [33]. Tetrahedral elements were adopted to better describe spherical particles. Mesh sensitivity tests with parametric models were carried out in [31] and meshes generated with voxel size of 0.25 mm were adopted.

Zero-thickness cohesive elements, representing ITZ, were inserted along the interfaces between aggregates and mortar phases by an in-house code. This was achieved by duplicating the exact node sets at the interface of aggregate and mortar. The original node set remains at the mortar continuum tetrahedral elements, while the duplicated one is attached to aggregate elements. Thus, 6-node cohesive elements with zero thickness (i.e., COH3D6 in ABAQUS) were generated by connecting the two node sets in 3D meso-scale concrete model.

## 4. Cohesive Zone Model and CDP Model

Zero-thickness cohesive element was briefly introduced in [31,34], and described in details in ABAQUS documentation [39]. To analyse the initiation of fracture, cohesive zone model was proposed [40,41,42], and extended to modelling concrete by Hillerborg et al. [43]. B-K criterion was proposed by Benzeggagh and Kenane [44] to evaluate mix mode delamination fracture toughness. In order to simulate mixed-mode fracture of composites, Camanho and Davila [45] developed a user-defined element subroutine in ABAQUS, which is the cohesive elements. For the current study, the bilinear traction-separation law of cohesive elements was used. Stiffness of cohesive elements was set to be $10^{5}$ MPa/mm, which was derived from the results of previous studies where it was found to range from $10^{4}$ to $10^{9}$ MPa/mm [12,14,23,46]. The other cohesive parameters are shown in Table 2. These were selected according to the following past works. The critical traction and fracture energy for normal mode were reported by López et al. [46] to vary between 2 and 3 MPa and between 0.01 and 0.1 N/mm, respectively. The ratio between properties in shear and normal modes were reported to range from 2 to 10 [12,14,23,46]. The effects of the parameters mentioned above on concrete behaviour in macro-scale is demonstrated in Section 5.3. The ITZ density was taken to be 2000 kg/m${}^{3}$ [12,37].

Concrete damage plasticity (CDP) model was adopted for mortar to describe its plastic-damageable behaviour under different loadings. The theory of CDP model is fully explained in ABAQUS user manual [39]. A brief description is introduced in this paper to discuss parameters selection. Lubliner et al. [47] proposed the CDP model, which was further extended by Lee and Fenves [48]. Concrete (or mortar) inelastic behaviour is the combination of isotropic tensile and compressive plasticity, and isotropic damage. When loading is applied, permanent plastic deformation occurs together with stiffness degradation due to damage accumulation. After peak stress is reached, softening response is observed. This constitutive model or other similar ones have been adopted in various loading conditions, e.g., monotonic, cyclic, and dynamic loading under low confining pressure [49,50,51].

Chinese Code GB50010 [52] provides the following expressions for the uniaxial tensile and compressive stress–strain relationships, the parameters of which are easily obtained from the tests. The tensile relation is linear elastic up to the peak stress, then follows Equation (1) in the post-peak region:

(1) $$\frac{\sigma_{t}}{f_{t}}=\frac{\frac{\varepsilon_{t}}{\varepsilon_{t0}}}{\alpha_{t}{\left(\frac{\varepsilon_{t}}{\varepsilon_{t0}}-1\right)}^{1.7}+\frac{\varepsilon_{t}}{\varepsilon_{t0}}}$$

where $\sigma_{t}$ is the current tensile stress, $\varepsilon_{t}$ is the current tensile strain, $\sigma_{t0}$ is the peak stress, $\varepsilon_{t0}$ is the corresponding strain, and $\alpha_{t}$ is a coefficient obtained by $\alpha_{t}=0.312\sigma_{t0}^{2}$ [53]. The full compressive relation is given by:

(2) $$\frac{\sigma_{c}}{f_{c}}=\left\{\begin{array}{c} \frac{E_{0}\varepsilon_{c}}{f_{c}},\;\frac{\sigma_{c}}{f_{c}}\leq 0.4\\ \alpha_{a}\frac{\varepsilon_{c}}{\varepsilon_{cu}}+\left(3-2\alpha_{a}\right){\left(\frac{\varepsilon_{c}}{\varepsilon_{cu}}\right)}^{2}+\left(\alpha_{a}-2\right){\left(\frac{\varepsilon_{c}}{\varepsilon_{cu}}\right)}^{3},\;\frac{\sigma_{c}}{f_{c}}\geq 0.4\;\&\;\frac{\varepsilon_{c}}{\varepsilon_{cu}}\leq 1\\ \frac{\frac{\varepsilon_{c}}{\varepsilon_{cu}}}{\alpha_{d}{\left(\frac{\varepsilon_{c}}{\varepsilon_{cu}}-1\right)}^{2}+\frac{\varepsilon_{c}}{\varepsilon_{cu}}},\;\frac{\varepsilon_{c}}{\varepsilon_{cu}}\geq 1 \end{array}\right.$$

where $\varepsilon_{cu}$ is the strain at damage initiation, $\alpha_{a}$ is a coefficient calculated by $\alpha_{a}=2.4-0.0125\sigma_{cu}$, and $\alpha_{d}$ is a coefficient given by $\alpha_{d}=0.157\sigma_{cu}^{0.785}-0.905$. The initial behaviour of concrete is linear elastic. This is followed by a brief hardening plasticity with limited damage until peak stress, and increasing damage leading to strain softening.

## 5. Results and Discussion

### 5.1. Models Calibration

The experimental and simulation results of concrete with 20%, 30% and 40% aggregate volume fractions are compared to investigate the effect of meso-structure obtained from image-based and parametric modelling. Cylindrical volumes with diameter of 50 mm and height of 100 mm are generated to reduce the computational cost. The same cylinders are used under tension. This is because direct tension test with cylinder is experimentally challenging. Therefore, dogbone specimens are used for direct tension tests.

Table 2 lists the parameters of aggregate, mortar and ITZ adopted in the current model. *E* denotes elastic modulus, ρ denotes density, ν denotes Poison’s ratio, $\sigma_{c}$ is the compressive strength, $\varepsilon_{c0}$ is the critical strain at peak stress, $\sigma_{t}$ denotes the peak tensile stress, k${}_{n}$ is the stiffness of cohesive elements, *t* and $G_{f}$ denote the critical strength and dissipation energy of cohesive elements, respectively. Linear elastic property is assigned to aggregate, where no transgranular failure is observed. The plastic-damageable behaviour of mortar is described with parameters obtained from compression tests of cylinder specimens and tension tests of dogbone specimens.It is noted that these mortar parameters are different from those used in the concrete with three aggregate contents. The ITZ parameters are obtained from the past studies and from calibration performed subsequently [12,14,23,46].

Displacement control is adopted to load all specimens from the direction parallel to cylinder axis prescribed at the two circular surfaces. Nodes at one surface are fixed to zero displacement, while those at the opposite surface are controlled by nodal displacements in tension, or by displacements from a rigid loading platen in compression. The friction between the rigid loading platen and the concrete sample is set to be 0.3 [34]. ABAQUS/Standard is applied to run all the simulation of concrete. Force-displacement curves are extracted to obtain stress–strain curves. The energy dissipation of mortar and ITZ is obtained from ABAQUS energy output, i.e., plastic (ELPD) and damage deformation (ELDMD). Regularisation by fracture energy can reduce the mesh size effects, but it should not be combined with viscoplastic regularisation [54]. In this work, fracture energy control of damage evolution is suitable for both damage-plasticity and traction-separation law [34].

### 5.2. Mesh Sensitivity Analysis

**Figure 5:**
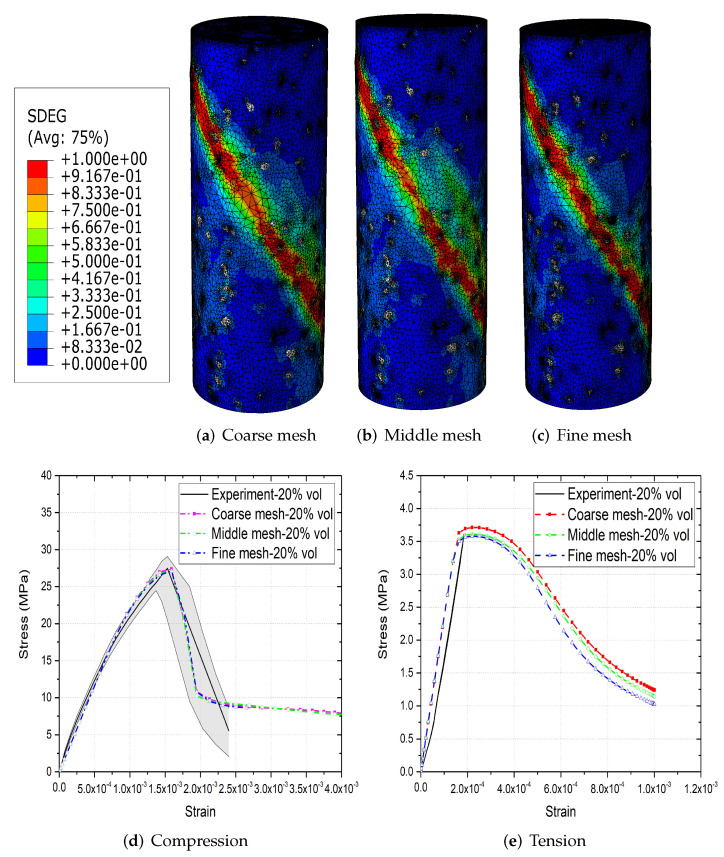
(**a**–**c**) Failure patterns of 20% vol image-based models with different meshes under compression and (**d**,**e**) their stress–strain curves under compression and tension.

Full mesh sensitivity results of parametric models can be found in a previous study by the authors [31]. The effect of mesh size on image-based concrete models is investigated in this section. Figure 5a–c show three different meshes of concrete model with 20% aggregate content. The effect of mesh size on concrete failure patterns under compression are observed to be insignificant. Similar is the results under tension. Figure 5d,e further illustrate that mesh size have negligible effect on stress–strain curves of image-based concrete models under both compression and tension. The coarse mesh has 393,157 nodes and 2,284,750 elements, while the fine mesh has 465,160 nodes and 2,707,451 elements. The CPU time of model with coarse mesh is much more efficient than fine mesh model, especially under compression [31]. In this case, models with coarse mesh are used for the following simulations. The same mesh quality is used for parametric models.

### 5.3. Image-Based vs. Parametric Meso-Structure

The stress–strain results, energy dissipation and damage patterns of meso-scale concrete obtained from XCT scan and generated synthetically, are analysed under compression and tension. The results suggest that the macro-scopic mechanical behaviour of both mesoscale concrete models subject to compression and tension has good agreement with experimental data, while the distribution of damage, rather than the dominant fracture pattern, between these models is the only substantial difference observed.

**Figure 6:**
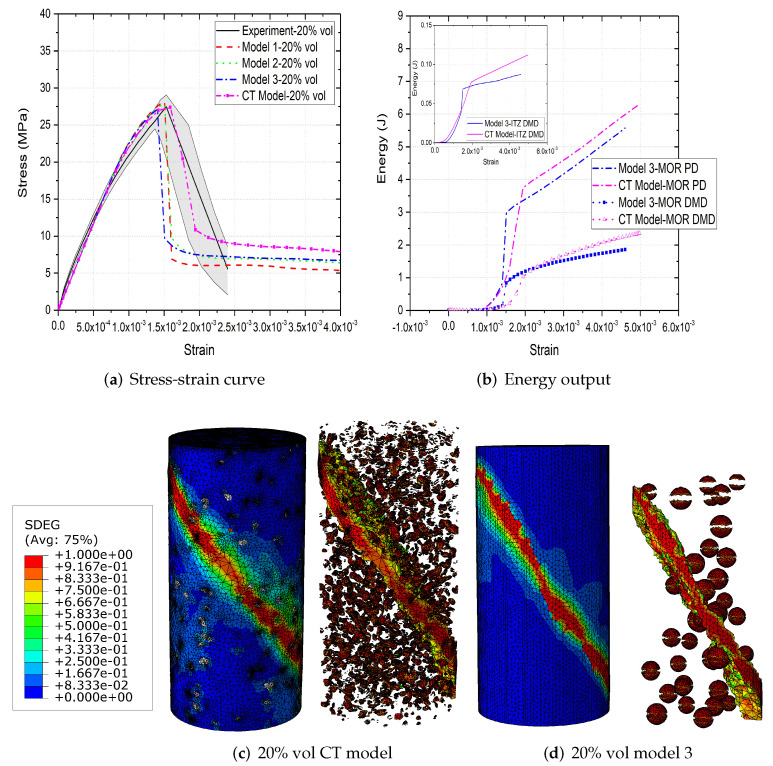
(**a**) Stress-strain curves, (**b**) energy output and (**c**,**d**) crack patterns of 20% vol parametric and CT concrete models under compression [31].

**Figure 7:**
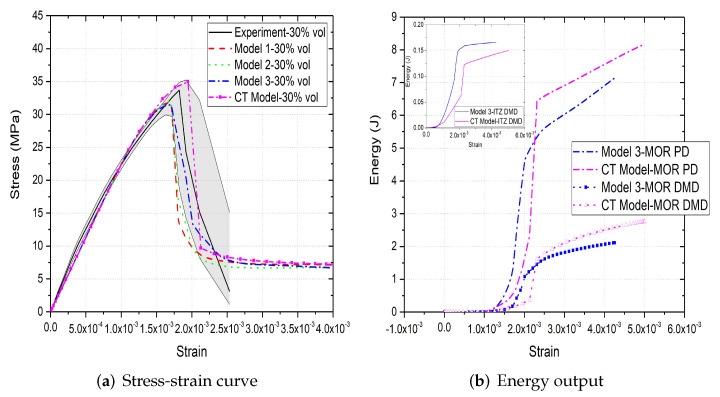

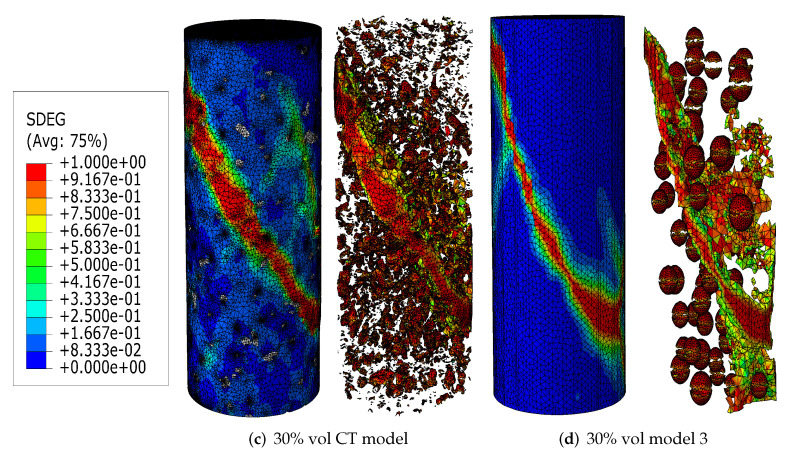
(**a**) Stress-strain curves, (**b**) energy output and (**c**,**d**) crack patterns of 30% vol parametric and CT concrete models under compression [31].

**Figure 8:**
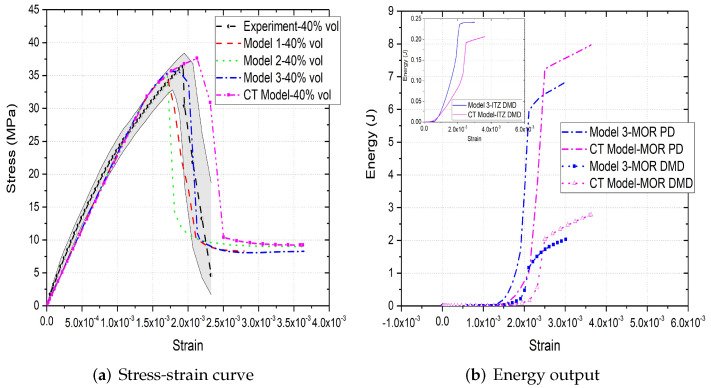

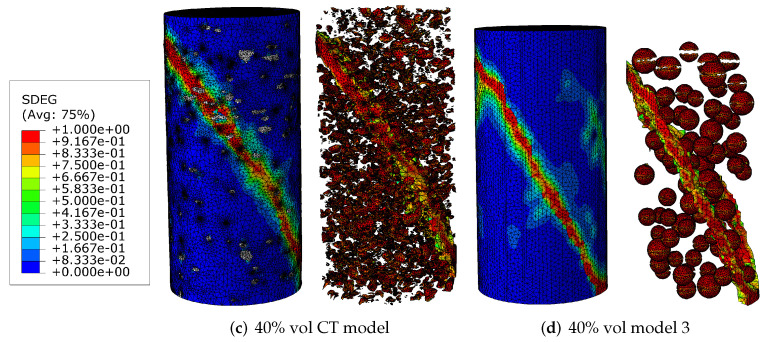
(**a**) Stress-strain curves, (**b**) energy output and (**c**,**d**) crack patterns of 40% vol parametric and CT concrete models under compression [31].

Figure 6a,b, Figure 7a,b and Figure 8a,b compare the compressive stress–strain results and energy dissipation of concrete with 20, 30 and 40% aggregate volume fractions, while Figure 6c,d, Figure 7c,d and Figure 8c,d illustrate the corresponding failure patterns. Note that the aggregates may seem to obstruct each other in a 2D image of a 3D model. Additionally, the real aggregates are not perfect spheres, but more close to ellipsoids or polyhedrons. Therefore, the number of aggregates in image-based meso-structure is larger than that in synthetic one with the same aggregate volume fraction, which leads to denser packaging of aggregates in image-based concrete model. However, the size distribution of both synthetic and natural aggregates matches the prescribed values. Both of the peak strength and critical strain at peak strength of image-based and parametric models have good agreement with experimental results in all concrete types. The pre-peak region of image-based model with 20% aggregate content agrees well with that of parametric model. However, the peak strength of image-based model is 11% and 6% larger than that of parametric model for 30% and 40% concrete, respectively, which is observed by [12,55], while the critical stain is 13% larger for both. One possible reason is that the natural aggregates have irregular shapes. Additionally, the post-peak softening region and residual stress of image-based and parametric models agree with each other.

The compressive failure patterns and damaged elements of image-based and parametric models with 20%, 30% and 40% aggregate content are shown in Figure 6c,d, Figure 7c,d and Figure 8c,d. The typical shear crack is observed, which is consistent with experimental observation in Figure 1c. The macro-cracks of both models are identical for all three types of concrete. The only substantial difference is the distribution of damage, especially for the cohesive elements of ITZ. This is because the aggregate size distribution and location in image-based model is more physically realistic compared with that in parametric model.

The inelastic behaviour of meso-scale concrete is represented by plastic dissipated energy (PD) of mortar and damage dissipated energy (DMD) of mortar and ITZ in current model. The energy dissipation of mortar and ITZ in 20% aggregate content image-based model is similar with that in parametric model. The only difference is that the energy dissipation rate of the former is less rapid than that of the latter, because the post-peak softening of the former is slower. The onset of plastic and damage dissipation of image-based model with 30% and 40% aggregate content is later than that of parametric model, because the critical strain of the former is larger. The energy dissipation rate is related with post-peak softening. For example, in concrete model with 30% aggregate, the rapid post-peak softening of image-based model is reflected on the rapid increase on dissipated energies of mortar and ITZ. Furthermore, more energy is dissipated through mortar plasticity and damage in image-based models with 30% and 40% aggregate compared with that in the corresponding parametric models. The reason is that the extra dissipated energy is required to achieve a higher peak strength in these image-based models. Finally, the ITZ damage dissipation of image-based models with 30% and 40% aggregate volume fractions is smaller than that of parametric models due to the smaller total surface area of ITZ in image-based model.

**Figure 9:**
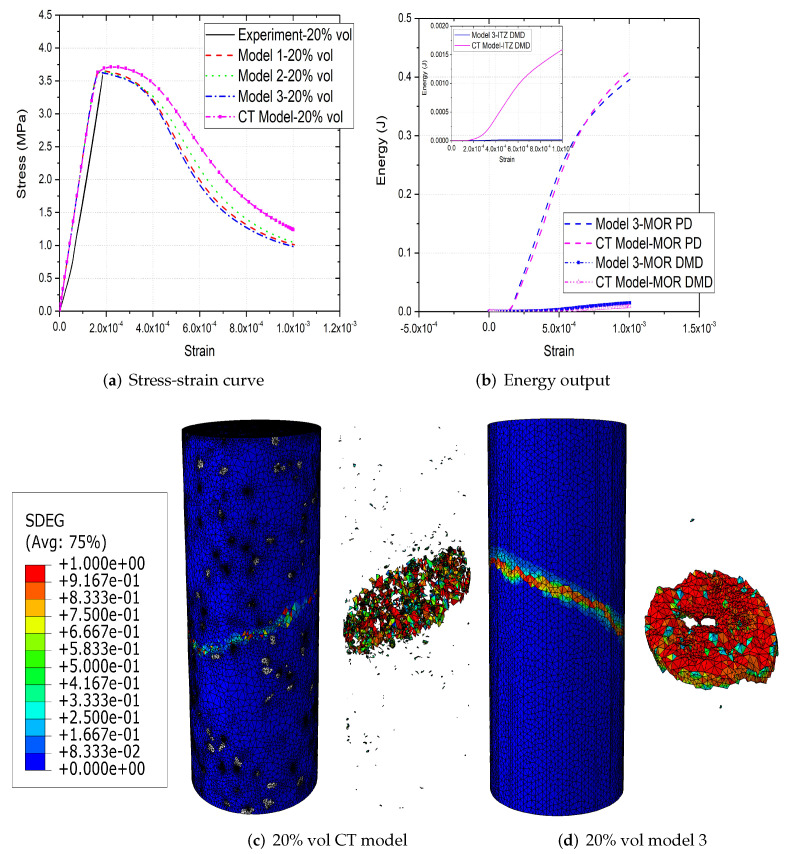
(**a**) Stress-strain curves, (**b**) energy output and (**c**,**d**) crack patterns of 20% vol parametric and CT concrete models under tension.

**Figure 10:**
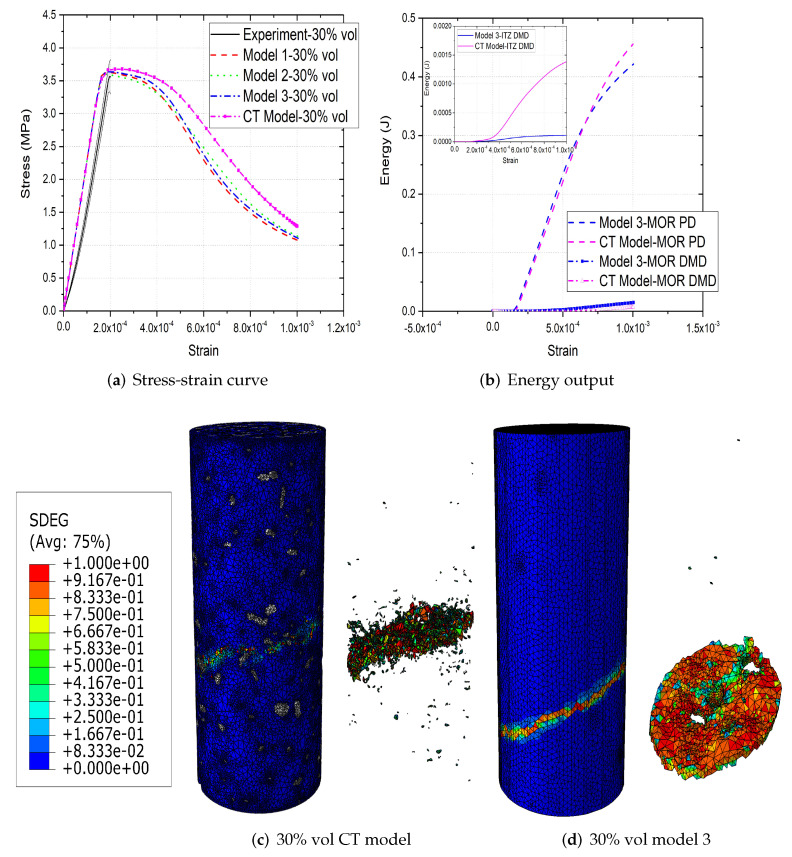
(**a**) Stress-strain curves, (**b**) energy output and (**c**,**d**) crack patterns of 30% vol parametric and CT concrete models under tension [31].

**Figure 11:**
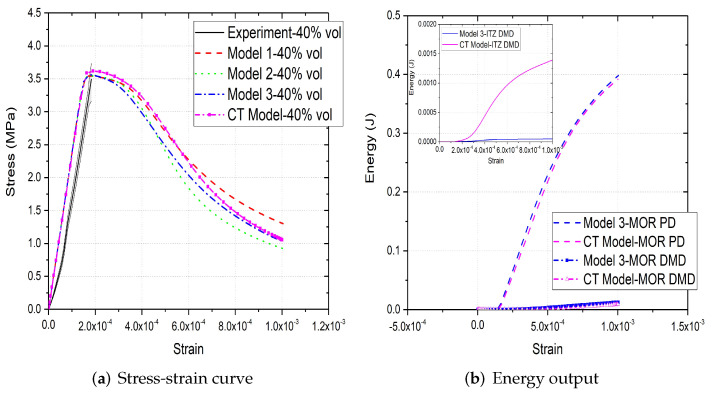

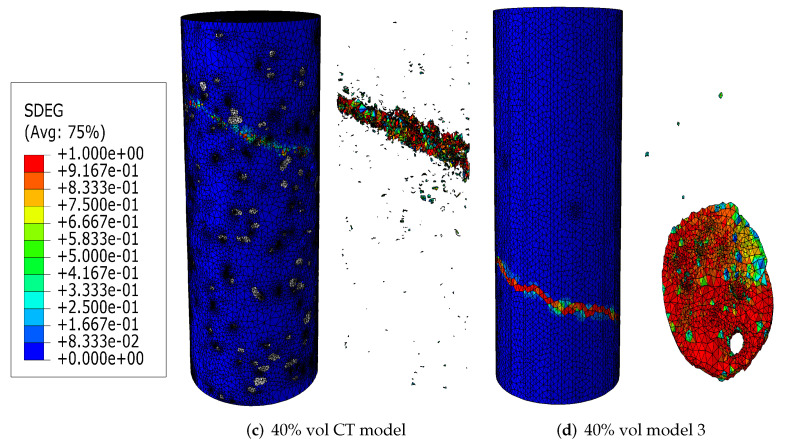
(**a**) Stress-strain curves, (**b**) energy output and (**c**,**d**) crack patterns of 40% vol parametric and CT concrete models under tension [31].

The tension results of concrete with 20%, 30% and 40% aggregate volume fractions are shown in Figure 9, Figure 10 and Figure 11. The peak tensile stress and its corresponding critical strain of image-based models have good agreement with that of parametric models for all three types of concrete. However, the post-peak softening of image-based model is slower than that of parametric model, which suggests that more energy is dissipated during the tension of image-based models. The mortar plastic and damage dissipation are the same for both models, while the ITZ damage dissipation of image-based model is ten times larger than that of parametric model. This results from the fact that more damaged ITZ elements are observed in image-based models for all three concrete types. The natural aggregates have more irregular shapes and stress concentration around the sharp corners compared with synthetic ones.

The concrete fracture energy in both models can be roughly estimated to be 203 J/m${}^{2}$, which is close to the total work of 0.4 J until failure and cylinder cross-section of 0.001963 m${}^{2}$. Similar value of fracture energy has been measured experimentally from the same type of concrete in [56].

As to tensile fracture patterns, one dominant crack is observed for concrete models. When aggregate content of concrete is low (i.e., 20% and 30%), the crack is located in the middle of cylinder. However, failure close to one end of cylinder is observed if concrete aggregate volume fraction is high, i.e., 40%.

Both parametric and image-based concrete models are in good agreement with experimental observations except the substantial difference in the distribution of damage. Note that the construction of both models needs to meet certain requirements. Self-developed code is required to generate randomly prescribed phase particles, while XCT scanning is expensive and image processing is time consuming.

### 5.4. General Discussion

Compared to image-based modelling, the advantage of the parametric modelling is that it can be used to generate meso-structures in larger material volumes while reproducing the basic statistical meso-structural characteristics - volume density and size distribution of aggregates. The size of the material volume is limited only by the computational resources. From a research perspective, the benefit of using material volumes larger than those that can be imaged is that they can be used to investigate size effects in concrete, arising from the ratio between the average aggregate size and a component size, and more generally to clarify the notion of representative volume element for a given concrete composition. From a more practical perspective, parametric meso-structures can be used to cover either whole structural elements or regions of such elements where damage is expected to initiate and propagate due to presence of stress concentrators. As a result, it can be used in advanced integrity assessments of critical components of engineering structures.

The advantage of image-based models is that they represent more realistically the shape, size, and spatial distribution of aggregates and pores. The benefit of this is that such models can be used to test different hypotheses regarding the constitutive behaviour of different phases, particularly when 4D imaging is used, i.e., when the scanning is performed during mechanical testing. A disadvantage is that the imaged volume is limited and there is no guarantee that the behaviour measured with such a volume is representative for a concrete specimen of a larger size. It has been demonstrated with the present work, that the selected constitutive behaviours for different phases with parameters calibrated by testing of mortar or selected from previous experience, lead to good predictions of the macroscopic stress–strain behaviour and damage evolution with both the image-based and the parametric models. This suggests that the imaged volumes have been close to representative for the concrete compositions studied.

## 6. Conclusions

Concrete specimens with 20%, 30% and 40% aggregate volume fractions are simulated with both image-based and parametric meso-structures under compression and tension. Stress-strain curves and failure patterns are analysed to assess the effect of aggregate distribution on concrete mechanical behaviour, while energy output is compared to explain the mechanisms. The main findings and recommendations of this work are:

- Artificial intelligent based segmentation tool, trainable Weka segmentation plugin of ImageJ, is effective and efficient in identifying various features of heterogeneous materials.
- Modelling meso-scale concrete inelastic behaviour with plastic-damageable mortar and damageable ITZ represented by zero-thickness cohesive elements is a practical and effective approach to deal with uniaxial compression, tension and potentially other complex loading conditions.
- The mechanical response of both image-based and parametric mesoscale models shows good agreement with compressive and tensile experimental data; differences between the two modelling approaches are observed only in the distributions of damage.
- Parametric models are recommended for analysis of size effects in concrete, as well as for modelling critical components or parts of such components in engineering structures.
- Image-based models are recommended for testing hypothesis for constitutive behaviour of concrete constituents, as well as for analysis of coupled behaviour, such as mass transport and deformation, where the realistic shape, size, and spatial distribution of constituents could have significant effect on the coupling.

## Figures and Tables

**Table 1:** The mix proportion design of concrete and mortar specimens [34].

Sample	w/c Ratio	Water (kg/m${}^{3}$)	OPC (kg/m${}^{3}$)	Sand (kg/m${}^{3}$)	6.3–10 mm Limestone
20% Concrete	0.49	230	469	1101	540
30% Concrete				831	810
40% Concrete				561	1080
20% Mortar	0.49	230	469	1101	-
30% Mortar				831	-
40% Mortar				561	-

**Table 2:** Parameters for CDP and cohesive zone models [31].

	Aggregate	Mortar			ITZ	
		20% Vol	30% Vol	40% Vol	Mode I	Mode II/III
E	45	24.1	21.4	20.2	-	-
ρ kg/m${}^{3}$	2700	2200			2000	
ν	0.2					
$\sigma_{c}$ MPa	-	36.3	49.7	56.5	-	-
$\varepsilon_{c0}$	-	2.61 × 10${}^{-3}$	3.55 × 10${}^{-3}$	4.17 × 10${}^{-3}$	-	-
$\sigma_{t}$ MPa	-	3.8	3.7	3.7	-	-
k${}_{n}$ N/mm${}^{3}$	-	-	-	-	1 × 10${}^{5}$	
*t* MPa	-	-	-	-	3.5	10.5
$G_{f}$ N/mm	-	-	-	-	0.03	0.09

**Table 3:** Mean peak stress and critical strain of experiment and simulation.

		Compression			Tension		
		20% Vol	30% Vol	40% Vol	20% Vol	30% Vol	40% Vol
Experiment	$\varepsilon_{c0}$	1.61 × 10${}^{-3}$	1.84 × 10${}^{-3}$	1.92 × 10${}^{-3}$	1.84 × 10${}^{-4}$	1.96 × 10${}^{-4}$	1.83 × 10${}^{-4}$
	$\sigma_{c}$ MPa	27.6	33.8	36.5	3.58	3.68	3.49
Parametric m.	$\varepsilon_{c0}$	1.38 × 10${}^{-3}$	1.71 × 10${}^{-3}$	1.88 × 10${}^{-3}$	1.76 × 10${}^{-4}$	1.76 × 10${}^{-4}$	1.76 × 10${}^{-4}$
	$\sigma_{c}$ MPa	26.7	31.5	35.5	3.63	3.63	3.57
Image-based m.	$\varepsilon_{c0}$	1.59 × 10${}^{-3}$	1.94 × 10${}^{-3}$	2.13 × 10${}^{-3}$	2.20 × 10${}^{-4}$	2.51 × 10${}^{-4}$	1.91 × 10${}^{-4}$
	$\sigma_{c}$ MPa	27.4	35.0	37.6	3.61	3.68	3.62

## Data Availability

The data presented in this study are available upon request from the corresponding author.
